# Plasticity Related Gene 3 (PRG3) overcomes myelin-associated growth inhibition and promotes functional recovery after spinal cord injury

**DOI:** 10.18632/aging.101066

**Published:** 2016-10-15

**Authors:** Thomas Broggini, Lisa Schnell, Ali Ghoochani, José María Mateos, Michael Buchfelder, Kurt Wiendieck, Michael K. Schäfer, Ilker Y. Eyupoglu, Nicolai E. Savaskan

**Affiliations:** ^1^ Department of Neurosurgery, Charité - Universitätsmedizin Berlin, D-10115 Berlin, Germany; ^2^ Brain Research Institute, University of Zurich and ETHZ, CH-8057 Zurich, Switzerland; ^3^ Art+Science Consulting, Zurich, Switzerland; ^4^ Translational Cell Biology and Neurooncology Lab, Department of Neurosurgery, University Medical School Erlangen, Friedrich Alexander University of Erlangen – Nürnberg (FAU), D-91054 Erlangen, Germany; ^5^ Center for Microscopy and Image Analysis, University of Zurich, CH-8057 Zurich, Switzerland; ^6^ Department of Anesthesiology, University Medical Center, Focus Program Translational Neuroscience (FTN) of the Johannes Gutenberg-University Mainz, Germany; ^7^ BiMECON Ent., Berlin, Germany; ^8^ Dr. Erler Kliniken Nürnberg, Nürnberg, Germany; ^9^ Present address: Department of Physics, University of California at San Diego, La Jolla, California 92093 USA

**Keywords:** myelin, neural cytoskeleton, axonal regeneration, plasticity, spinal cord injury: rehabilitation

## Abstract

The Plasticity Related Gene family covers five, brain-specific, transmembrane proteins (PRG1-5, also termed LPPR1-5) that operate in neuronal plasticity during development, aging and brain trauma. Here we investigated the role of the PRG family on axonal and filopodia outgrowth. Comparative analysis revealed the strongest outgrowth induced by PRG3 (LPPR1). During development, PRG3 is ubiquitously located at the tip of neuronal processes and at the plasma membrane and declines with age. *In utero* electroporation of PRG3 induced dendritic protrusions and accelerated spine formations in cortical pyramidal neurons. The neurite growth promoting activity of PRG3 requires RasGRF1 (RasGEF1/Cdc25) mediated downstream signaling. Moreover, in axon collapse assays, PRG3-induced neurites resisted growth inhibitors such as myelin, Nogo-A (Reticulon/RTN-4), thrombin and LPA and impeded the RhoA-Rock-PIP5K induced neurite repulsion. Transgenic adult mice with constitutive PRG3 expression displayed strong axonal sprouting distal to a spinal cord lesion. Moreover, fostered PRG3 expression promoted complex motor-behavioral recovery compared to wild type controls as revealed in the Schnell swim test (SST). Thus, PRG3 emerges as a developmental RasGRF1-dependent conductor of filopodia formation and axonal growth enhancer. PRG3-induced neurites resist brain injury-associated outgrowth inhibitors and contribute to functional recovery after spinal cord lesions. Here, we provide evidence that PRG3 operates as an essential neuronal growth promoter in the nervous system. Maintaining PRG3 expression in aging brain may turn back the developmental clock for neuronal regeneration and plasticity.

## INTRODUCTION

Neuronal plasticity and structural remodelling are fundamental feature of the developing nervous system and plays also an essential role during learning and injury-dependent remodelling and regeneration [1]. In development, axons extend over long distances and form contacts with their target structure and facilitate functional connections [2]. These neuronal connections become stabilized and restricted during maturation and secure proper functioning of the brain [1]. Conversely, sprouting and regeneration is limited after decline of intrinsic axonal remodelling activity in aging brain and in an microenvironment rich in neurite growth inhibitors after neurological injury [3].

Several extracellular ligands account for the neurite growth inhibitory environment after maturation and injury [3]. One such factor represents lysophosphatidic acid (LPA) which is a bioactive lipid borne from astrocytes and blood serum [4–6]. Another blood inherent factor with neural shape forming activity is thrombin and its receptor-activating peptide domain (TRP). Nogo-A/RTN4 (Reticulon-4) is a myelin-associated inhibitor derived from oligodendrocytes found to execute neurite growth inhibitory and collapsing activity in the adult [7, 8]. Although LPA, TRP and Nogo-A act on different receptors (LPA on LPA1-5 receptors; TRP on thrombin receptor and Nogo-A on NgR and PirB/LILRB2), all ligands converge on RhoA -Rho kinase pathway mediating the final signal transduction for neurite retraction and axon growth inhibition [9]. Concomitantly, recent landmark studies revealed that pharmacological and genetic interfering with Nogo/NgR function or LPA signaling promotes axonal regeneration and functional recovery after CNS injury [10, 11].

An essential step during development and regeneration is the initiation of actin-rich membrane protrusions termed filopodia or microspikes. These structures are involved in cell attachment, migration and neurite growth [12]. Filopodia initiation and neural growth depends on cytoskeletal dynamics regulated to a large extent by the small molecular weight GTPases of the Rho family. In particular, the Rho family GTPase Cdc42 regulates bundled actin filaments extending from the cell periphery, forming canonical filopodia of different length and width [13]. Historically, filopodia initiation research focused around the activation of WASP and N-WASP by Cdc42 [14]. The interaction of Cdc42 with WASP and N-WASP in the presence of phosphatidylinositol-4,5-bisphosphate (PIP2) leads to activation of the ARP2/3 complex which acts as a nucleator and growth promoter for actin filaments. However, Cdc42-independent filopodia formation also exists. However, WASP and N-WASP is often not present in filopodia and neurite growth occurs even in the absence of N-WASP [15, 16]. Moreover, depletion of upstream Cdc42 or downstream ARP2/3 leaves filopodia growth unaffected [17, 18]. The recently discovered formins, known as actin regulatory proteins located at the tips of filopodia indicates an alternative growth pathway [19, 20]. Formins act independently of ARP2/3 and recruit the GTPase Rif (Rho in filopodia) instead of Cdc42. These molecules are critically involved in neurite growth control and are implicated to overcome neurite growth inhibition signals [21]. Interestingly, PRG3 acts independently of Cdc42 and VASP family proteins, pointing to a recently discovered role of integral membrane proteins in the shaping of membrane curves [22].

Here, we describe the individual morphogenic activity of the integral membrane proteins Plasticity Related Genes also termed Lipid Phosphate Phosphatases Related genes (PRG 1-5 or LPPR 1-5). They are differentially expressed in the developing brain and re-expressed in regenerating axons after a lesion [11, 23]. In particular, PRG3 induces the formation of filopodia and promotes axonal growth [23]. The sequence of PRG3 is highly related to PRG5 which also promotes morphological changes in neurons [24]. However, our comparative analysis revealed a hierarchy with PRG3 displaying the strongest outgrowth promoting activity among the entire PRG family.

We characterized the required PRG3 domains for filopodia inducing activity and found that the localization of the carboxyl terminal is critical for neurite growth. Moreover, PRG3 overcomes neurite retraction of common brain and blood borne neurite growth inhibitors such as Thrombin, Nogo-A and LPA *in vitro* and *in vivo*.

## RESULTS

### PRG family members change neuronal cell shape differently

**Figure 1:**
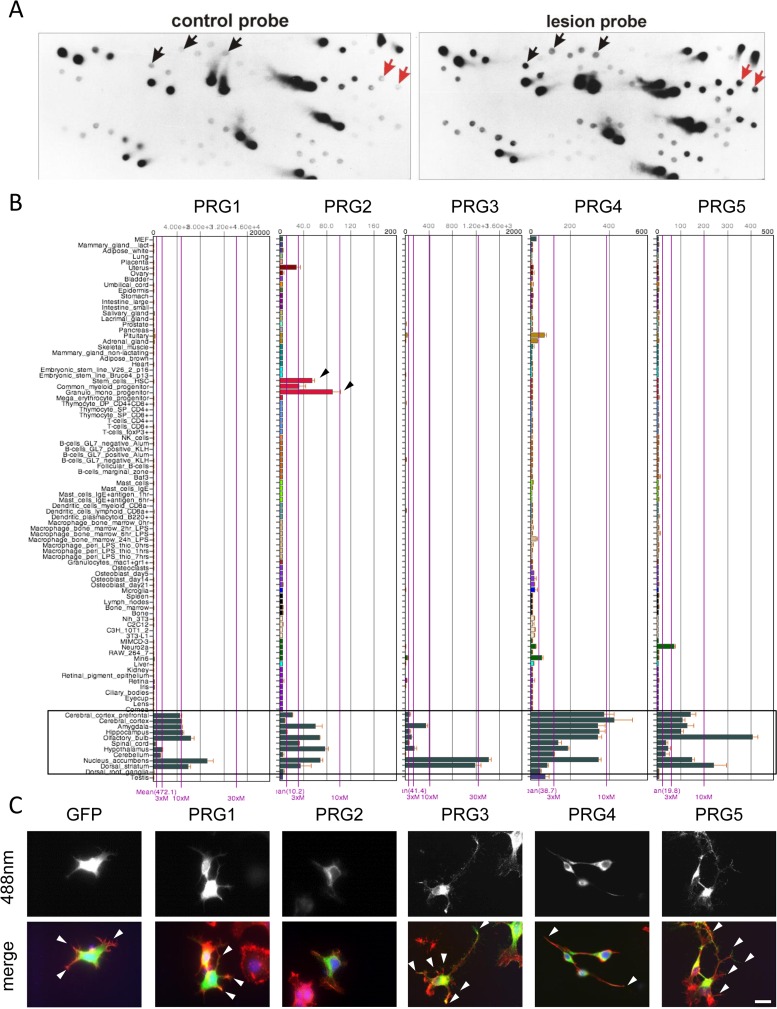
Differential subtractive cDNA library screening reveals Plasticity-Related genes (PRGs) with neuronal cell morphology challenging effects (**A**) Representative differential subtractive cDNA dot blot library screening for lesion-induced genes in the hippocampus. Duplicative dot blots were probed with cDNAs from controls (adult hippocampus cDNAs, left) and lesioned brains (differentiated hippocampus cDNAs, right). Note that some genes are solely detectable with probes from lesioned hippocampus (red arrows), whereas others are quantitatively regulated (black arrows). (**B**) BIO-GPS organ and tissue expression analysis of PRG1-5 (left to right) in the mouse. Note, that expression of PRGs is preferably high in different brain areas (black framed box). PRG1 is expressed highest with an average of 472.1 and PRG2 is lowest with 10.1 in average (average compared to overall mean expression of individual array). Notably, PRG2 is also expressed in myeloid progenitors (black arrowheads). (**C**) Comparative analysis of GFP and various PRG family members in (GFP and PRG1 to 5-GFP, left to right) in murine neuronal cells N1E-115. Overexpression of PRG3 and PRG5 in particular enhanced filopodia formation and neurite outgrowth (white arrowheads). Expression of PRG1 and PRG2 do not challenge the neuronal phenotype compared to controls. PRG4 induces a bipolar phenotype (arrowheads). Scale bar represents 20 μm.

We described previously that a subtraction hybridization screen and degenerative PCR primer search led to the identification of the Plasticity Related Gene family (PRG) (Fig. 1 A) [11, 23, 24]. Sequence alignments of all five PRG members revealed high homology in all six transmembrane domains. However, the carboxy terminus (CT) located domain varies significantly among the PRG members with high sequence similarity between PRG3 and PRG5 (Fig. S 1). Human PRG3 amino acid sequence shares 57 % homology to PRG5 indicating it as its closest relative within the PRG family. Structural searches predict three extracellular domains D1-D3, also conserved in a subset of the type 2 phosphatidic acid phosphatase ectoenzyme superfamily (prototypic lipid phosphate phosphatase 1 and 3, (LPP1, LPP3)) (Fig. S 1). The five PRG family members identified in the human genome differ in tissue distribution and expression (Fig. 1 B). The expression of individual PRGs varies particularly in cerebral cortex, olfactory bulb, and nucleus accumbens among each other (Fig. 1 B).

We analysed the distribution of the PRG family members and their impact on neuronal cell morphology. PRG proteins were localised at the plasma membrane of neuronal cells, but only PRG3 and PRG5 displayed enriched distribution at the tips of neurites and microspikes (Fig. 1 C). Moreover, solely PRG3 and to a lesser extent PRG5 caused massive neurite and filopodia growth (Fig. 1 C). On the other hand, PRG4 induced a bipolar spindle-like phenotype and PRG1 and PRG2 marginally altered cellular morphology compared to GFP expressing control cells (Fig. 1 C).

We performed automated cell phenotype analysis with a modified version of the publicly available neuronal outgrowth pipeline (CellProfiler 2.0, Broad Institute Harvard/MIT). Three parameters covering neuronal tree complexity were analysed:

1. number of trunk branches, indicating branches directly connected to the cell body (soma);
2. number of branch ends, labelling branch tips and
3. number of non-trunk branches, peripheral neurite connections neither directly connected to the soma nor to branch ends.

First, we identified a significant increase of trunk-associated neurites in PRG3-expressing neurons which was strongest compared to other PRG family members (Fig. S 1 B). Second, the number of secondary and tertiary branches was also significantly increased. Finally, PRG3 expression multiplied the number of branch ends compared to controls (Fig. S1 C).

### *In vivo* neuronal morphology shape by PRG3

**Figure 2:**
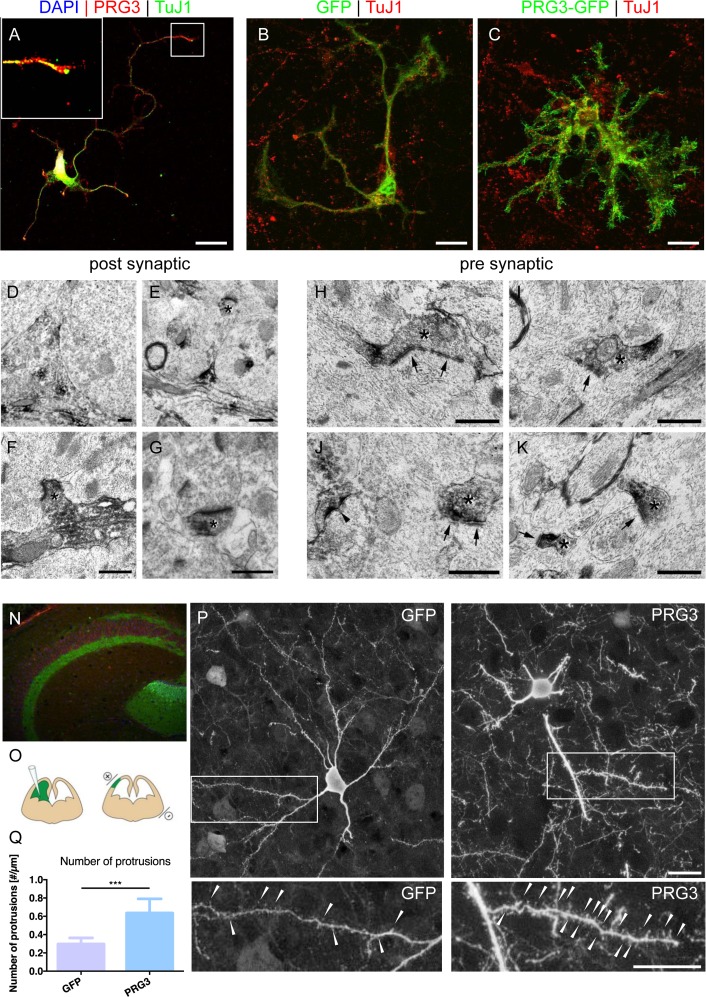
PRG3 is located at pre-synaptic domains *in vitro* and *in vivo* (**A**) Endogenous PRG3 (red colour) is located at the plasma membrane and accumulated in TuJ1 positive axonal processes (green) in rat cortical neurons. Scale: 20 μm. (**B**) GFP vector transfection of rat primary cerebellar granular cells does not significantly alter cell morphology. TuJ1 co-staining shows neuronal origin (red). Scale: 20 μm (**C**) PRG3 overexpression (green) in primary cerebellar granular cells induces and accelerate neuronal outgrowth compared to controls (GFP, green). TuJ1immunostaining is given in red. Scale: 20 μm. (**D-G**) Pre-embedding electron microscopy immunolocalization of PRG3. Subcellular analysis reveals post-synaptic distribution of PRG3. DAB accumulated in the cytoplasm (**D**). Dendritic spine structures were also found positive for PRG3 and are indicated by asterisks (**E-G**). Scales: 0.5 μm. (**H-K**) Occasionally, axonal boutons (pre-synaptic structures), appeared labeled with PRG3 antibodies (asterisk). Accumulations of DAB appeared especially in the pre-terminal parts of the axonal boutons. Postsynaptic elements are marked with arrows and a labeled spine with an arrowhead in J. Scales: 0.5 μm. (**N**) Immunohistochemical staining identifies endogenous PRG3 expression (green) is located primarily in the hippocampus in the adult mouse brain (Red = TuJ1). (**O**) Scheme of the experimental protocol for cortical *in utero* electroporation [55]. (**P**) Representative example of electroporated brain section showing pyramidal neurons positive for GFP expressing pyramidal neurons (left) and PRG3-positive pyramidal cells (right). Neuronal morphology was analysed at postnatal day 10 (P10). Scale bar represents 20 μm. High-power magnifications of boxed areas show spines and spine-like membrane protrusions, which are indicated by arrowheads. Scale bar represents 20 μm. (**Q**) Number of protrusions per μm dendrite were quantified in 70 μm confocal stacks. Neurons electoroporated with PRG3 show significantly more protrusions per μm compared to GFP electroporated neurons. Values are given as mean ± SEM. (N=5). Statistical analysis was performed using two tailed student's t-test. P value was set as * = p<0.05: ** = p<0.01; *** = p<0.001.

We further investigated PRG3 location *ex vivo* and found it expressed in axon tips of primary neurons (Fig. 2 A). Endogenous PRG3 was located at the tip of actin-rich growth cones of cortical neurons (Fig. 2 A; Fig. S 2). Interestingly, primary astrocytes were almost immuno-negative for PRG3 (Fig. S 2). To investigate whether PRG3 has a general impact on neuronal morphology independently of the type of neurons, we studied this gene in cerebellar neurons. PRG3 expression in rat granule neurons caused extensive formation of neurites and filopodia in comparison to GFP expressing control granule neurons (Fig. 2 B, C). Electron microscopy studies of hippocampal synapses revealed post-synaptic (Fig. 2 D-G) and occasional pre-synaptic location of PRG3 (Fig. 2 H-K). Immuno-histochemistry of brain cryo-sections identified hippocampal neurons with high PRG3 levels in the adult mouse brain (Fig. 2 N).

For *in vivo* assessments we performed *in utero* electroporation of mouse embryonic cortical neurons at embryonic day 13 (Fig. 2 O) with GFP control and PRG3 constructs (Fig. 2 P). Noteworthy, neonates survived the procedure without obvious constraints and were sacrificed at postnatal day 10 (P10). In depth morphometric investigations of single pyramidal neurons displayed a higher protrusion density of PRG3 positive neurons. These data demonstrate that PRG3 operates on neural shape and filopodia in vivo (Fig. 2 P).

### PRG3 C-terminal domain promotes neurite growth and branching

PRG3 and PRG5 are both the smallest PRG family members with the shortest intracellular c-terminal (CT) domains of 46 and 47 amino acids, respectively (Fig. S 1 A). We hypothesized, that the unique CT domain of PRG3 which is absent in other PRG family members, might be causal for the enhanced differentiated neuronal phenotype.

**Figure 3:**
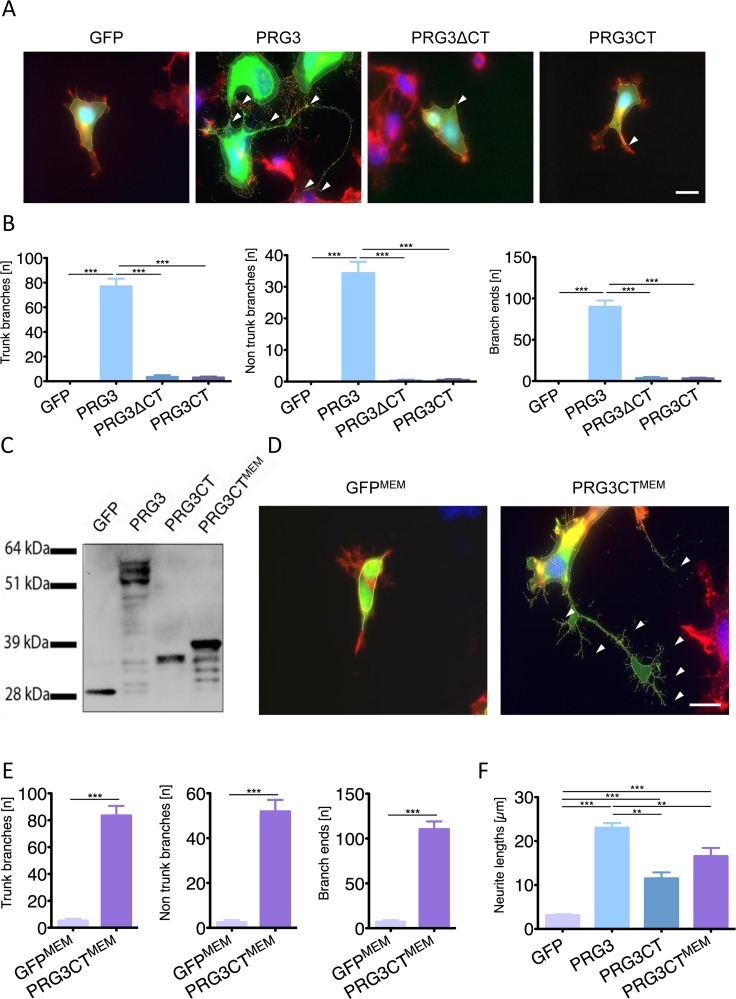
Plasma membrane localization of the PRG3 C-terminal domain is essential for axon outgrowth (**A**) PRG3 overexpression induces neurite outgrowth (PRG3, arrows) in comparison to controls (GFP). A truncated PRG3 construct of its C-terminal domain (PRG3ΔCT) and a truncated PRG3 construct consisting of solely the C-terminus (PRG3CT) was compared. Expression of PRG3ΔCT and PRG3CT induces solely a few neurites comparable to controls (arrowheads). Scale bar represents 20 μm. (**B**) Quantification of trunk, non-trunk branches and branch ends in controls (GFP), PRG3, PRG3ΔCT and PRG3CT. Data are given as mean ± SEM from three independent experiments. Statistical differences were analysed by one way anova with Bonferroni post hoc analysis. P value was set as * = p<0.05: ** = p<0.01; *** = p<0.001. (**C**) Total cell lysates from neuronal cells transfected with GFP or GFP fusion constructs, PRG3, PRG3CT, and PRG3CT^MEM^ were examined by immunoblotting using GFP-specific antibody. PRG3CT^MEM^ shows slight protein weight gain compared to PRG3CT due to the membrane myristoylation consensus tag (YES). (**D**) Comparison of membrane associated GFP (GFP^MEM^) and PRG3CT^MEM^ in neuronal cells. Note the increase in neurites and filopodia in PRG3CT^MEM^ expressing neurons compared to GFP^MEM^ controls (arrowheads, scale bar shows 20 μm). (**E**) Quantification of axonal branches, non-trunk branches and branch ends of GFP^MEM^ and PRG3CT^MEM^ expressing neurons. Values are given from three independent experiments and statistical analysis was performed using two tailed student's t-test. Error bars are given as ± SEM from three independent experiments. of each group. (**F**) Comparison of total neurite length between different expression clones. Statistical analysis was performed one way anova with Bonferroni post hoc analysis for multiple comparisons; * p<0.05, ** p<0.01, *** p<0.001. Error bars are given as ± SEM of each group.

To investigate this further, we generated a PRG3 construct lacking the CT domain (PRG3ΔCT) and another mutant construct with solely the CT domain (PRG3CT). Both constructs eliminated the effect induced by wild-type PRG3 (Fig. 3 A). We found the overexpressed CT domain primarily in the cytosol, whereas in the wild-type situation the CT domain is located at the plasma membrane. Hence, we fused the myristoylation consensus sequence of the YES-kinase together with the PRG3CT sequence to generate a membrane-targeted PRG3CT fusion protein (PRG3CT^MEM^, Fig. 3 C). The PRG3 phenotype was recovered when PRG3CT^MEM^ was expressed with respect to number of trunk branches, non-trunk branches and branch ends (Fig. 3 D, E). Neurite length measurements of GFP, PRG3CT^MEM^ and PRG3 revealed PRG3CT^MEM^ neurites grew significant longer compared to PRG3CT mutants and controls (Fig. 3 D, E). Thus, the subcellular localization and final position of PRG3CT is significantly linked to the functional neurite and filopodia growth promotion activity.

Serving as a control we cloned a YES-GFP construct (GFP^MEM^) to monitor the influence of YES-kinase domain on cellular morphology (Fig. 3 D). In fact, GFP^MEM^ neuronal shape and neurite processes were comparable to wild-type GFP controls (Fig. 3 D).

Furthermore, we tested different domains of PRG3 in terms of neurite promoting activity. PRG3CT^MEM^ enhan-ced neurite length similar to the level of the PRG3 wild-type (Fig. 3 F). The impact of PRG3 and its CT domain on cell morphology was more generally assessed in HeLa and embryonic epithelial P19 cells. HeLa cells displayed a minor although significantly increased number of trunk branches, non-trunk branches and branch ends in the case of PRG3 expression (Fig. S3 C-D). No significant increase was found after expression of PRG3CT^MEM^ (Fig. S3 C-D). Endogenous PRG3 mRNA was almost absent in undifferentiated P19 cells (Fig. S4 B). Noteworthy, retinoic acid differentiated P19 cells formed neurite-like outgrowth and the same time showed high PRG3 mRNA levels (Fig. S4 B). PRG3 transfection of undifferentiated P19 cells significantly increased neurite-like extensions and filopodia growth (Fig. S3 A-B). No difference was found in PRG3CT^MEM^ expressing cells.

### PRG3 requires RasGRF1 for axonal outgrowth and filopodia induction

**Figure 4:**
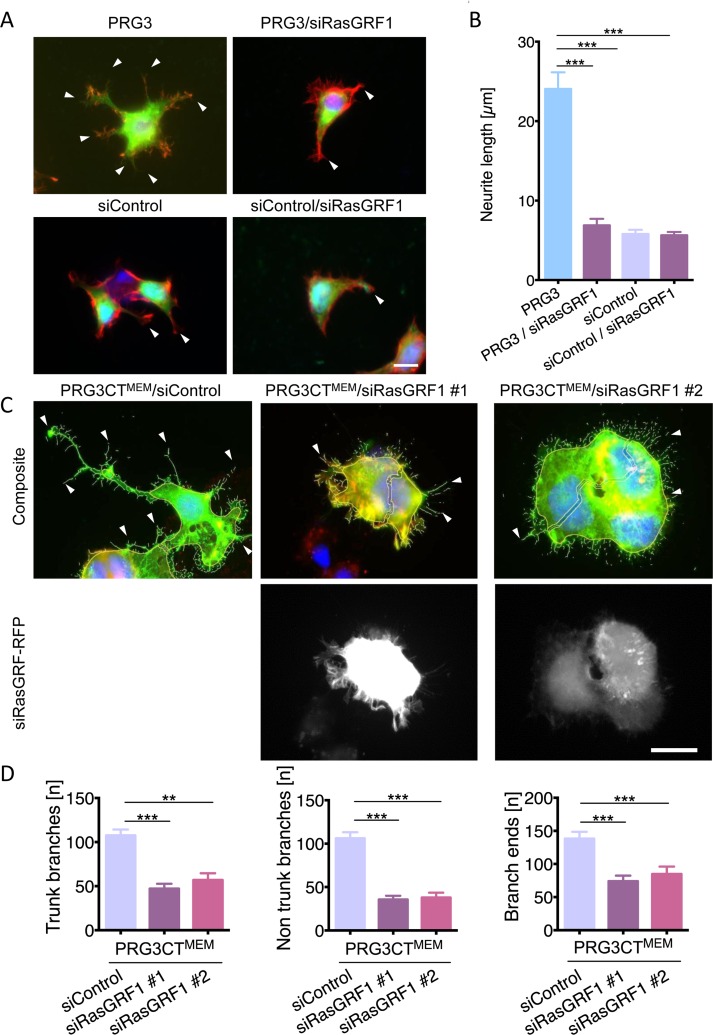
RasGRF1 executes PRG3-induced neuronal sprouting and elongation (**A**) Representative images of siRNA mediated knockdown of RasGRF1 and PRG3. Top panel, PRG3 transfected cells show massive neurite sprouting (arrowheads, left). siRNA mediated knockdown of RasGRF1 in PRG3 overexpressing cells (PRG3/siRasGRF1) show reduced sprouting and membrane protrusions (arrow, right). Bottom panel, siRNA mediated knockdown of RasGRF1 (right) does not affect the morphology of controls (scrambled siRNA-transfected cells, left, arrows). Scale bar gives 20 μm. (**B**) Quantification of neurite length in neurons overexpressing PRG3 (PRG3), PRG3 overexpressing and silenced RasGRF1 (PRG3/siRasGRF1), control transfected (siControl) and control transfected and RasGRF1 siRNA knockdown (siControl/siRasGRF1). Statistical differences are calculated from three independent experiments tested with one way anova with Bonferroni post hoc analysis for multiple group comparisons. Values are given as mean ± SEM, * p <0.05, ** p<0.01, *** p<0.001. (**C**) Representative images of control shRNA (siControl) and two different RasGRF1 RFP-shRNA knockdown vectors (siRasGRF #1 and siRasGRF #2) in PRG3CT^MEM^ expressing neurons show reduced filopodia formation and neurite outgrowth (arrowheads). Scale bar represents 20 μm. (**D**) Quantification of neuronal morphology in PRG3CT^MEM^ expressing and siRNA mediated RasGRF1 knockdown cells. Neurons were analysed for number of branches at the soma (trunk branches), peripheral branches (non-trunk branches), and tip structures (branch ends). Differences were considered statistically significant with * p<0.05, ** p<0.01, *** p<0.001 (one way anova, Bonferroni post hoc analysis for multiple comparisons). Values are given as mean ± SEM.

We identified potential PRG3 binding candidates in a yeast two-hybrid screen using the C-terminal tail of PRG3 as bait. Thereby, RasGRF1 (also termed GRF1/ RasGEF1/ CDC25) was discovered as a potential binding partner of PRG3. Follow-up binding studies revealed that PRG3 interacts with RasGRF1 [25]. To further proof the functional validity of this interaction, we introduced functional RasGRF1 siRNAs (Fig. S4 A). Reduction of RasGRF1 in PRG3 overexpressing clones significantly impaired the neurite and filopodia outgrowth phenotype found with PRG3 alone (Fig. 4 A, B). Moreover, RasGRF1 silencing resulted in neutralizing effects of PRG3CT^MEM^ in neurons (Fig. 4 D). Trunk branches and branch ends were reduced to control levels in PRG3CT^MEM^ neurons when RasGRF1 was depleted (Fig. 4 D). These data indicate that PRG3CT requires RasGRF1 to operate on filopodia and neurite growth.

### PRG3 modulates RhoA-Rock-PIP5K induced signaling

**Figure 5:**
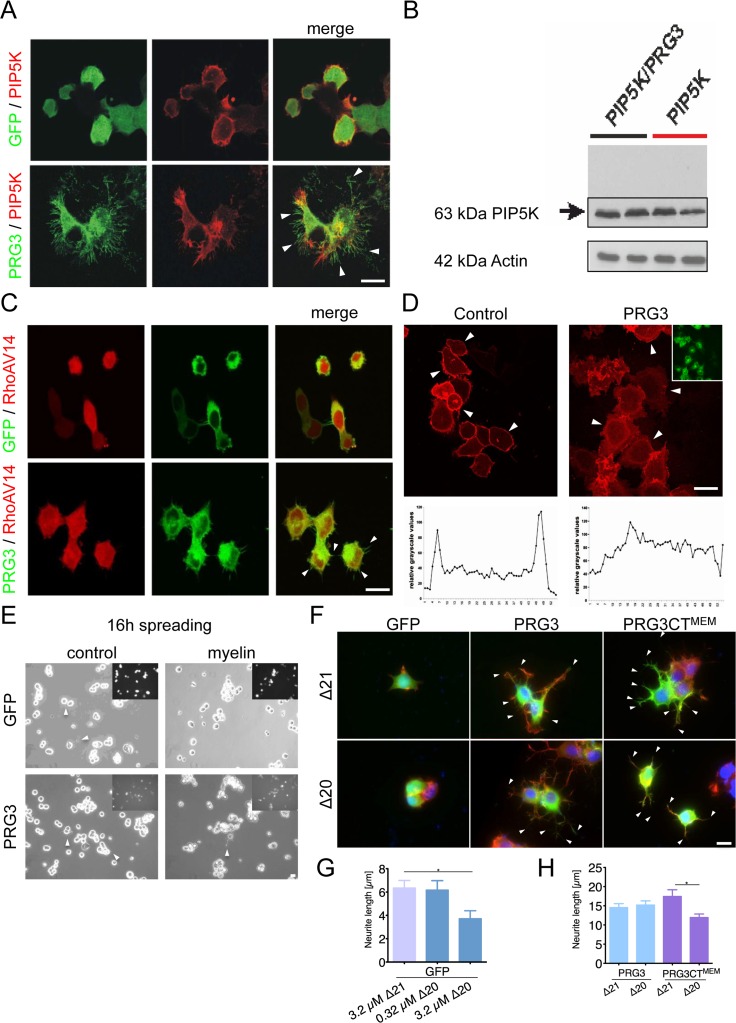
PRG3 impedes myelin and RhoA-induced axon collapse and translocates PIP2 from the plasma membrane (**A**) Representative images of neurons expressing GFP and PIP5K (control) or PRG3 and PIP5K (PRG3). PRG3-expressing neurons still retain their complex morphology in the presence of PIP5K overexpression (arrows). Scale bar represents 20 μm. (**B**) Immunoblot for PIP5K expression in PRG3 expressing neurons. Bottom, Actin serves as a house keeping protein for equal gel loading. (**C**) Representative images of co-overexpression of RFP and PRG3 in the presence of dominant active RhoAV14. Note the increased filopodia in PRG3 expressing neurons (arrows). Scale bar represents 20 μm. (**D**) Disruption of phosphoinositol-(4,5)-bisphosphate (PIP2) in PRG3 expressing neurons. Representative pictures of GFP (control) and PRG3 overexpressing (PRG3) neurons expressing the RFP – PLC1 PH domain fusion constructs (red) as an in vivo probe for intracellular PIP2 localization. Transfection efficacy is given in the upper right corner. Bottom, representative trans-cellular pixel traces of PIP2 values in GFP (control) and PRG3 overexpressing (PRG3) neurons. In PRG3 cells PIP2 membrane dislocation could be observed (arrows). Scale bar represents 20 μm. (**E**) Axon spreading assay on myelin-coated substrates. Representative images of a spreading assay with GFP and PRG3 overexpressing neurons on control substrate and myelin substrate. Note that PRG3 expressing neurons form neurites after 16 hours on myelin. (**F**) Representative images of control (GFP), PRG3 and PRG3CT^MEM^ cells treated with 3.2 μM Delta 20 (Δ20, bioactive neuronal contraction domain of Nogo-A). Neuronal collapse was detected in controls and PRG3CT^MEM^ expressing cells compared to treatment with Δ21 (control peptide sequence of Nogo-A without collapse activity). (**G**) Quantification of neurite length of GFP expressing neurons. Three independent experiments were carried out and differences were considered statistically significant with * p<0.05, ** p<0.01, ***p<0.001 (two-sided *t-*test). Values are given as mean ± SEM. (**H**) Quantification of neurite length of PRG3 and PRG3CT^MEM^ expressing neurons. Δ20 had no effect on PRG3 overexpression neurons but significantly reduced neurite length of PRG3CT^MEM^. Three independent experiments were carried out and differences were considered statistically significant with * p<0.05, ** p<0.01, ***p<0.001 (two-tailed *t-*test). Values are given as mean ± SEM.

Most myelin-derived neurite growth inhibitors execute their effects directly or indirectly through the RhoA-Rho-PIP5K pathway [26]. RhoA activates its downstream target PIP5K, which operates on cyto-skeleton reorganization [27, 28]. Interestingly, PRG3 rescued the round phenotype induced by PIP5K, without affecting PIP5K expression levels (Fig. 5 A, B). This indicates that PRG3 can counteract the neurite growth inhibitory action of PIP5K rendering it as a potential target for therapeutic interventions. We challenged the experiment with the dominant active PIP5K upstream effector RhoA^V14^. RhoA^V14^ induced neurite collapse in control neurons as described previously [24]. Co- expression of PRG3 and RhoA^V14^ still revealed neurite growth and filopodia formation although neurite length was reduced (Fig. 5 C). Further upstream, plasma membrane bound PIP_2_ regulates activation of RhoA and PIP5K [29, 30]. Therefore, we analyzed PIP_2_ distribution after PRG3 overexpression. These assays revealed that the ratio cytosolic fraction vs. membrane bound PIP_2_ was altered after PRG3 expression (Fig. 5 D). Conclusively, PRG3 over-expressing neurons resist neurite retraction by modulating RhoA activity via PIP_2_ membrane translocation.

### PRG3 neutralizes myelin-growth inhibition

The potential of PRG3 to overcome RhoA induced neuronal collapse led us to assess the functional implications of PRG3 expression in neurons. We determined neuronal spreading activity on myelin substrates, a major neurite growth inhibitory substrate present in spinal cord injured tissue [7, 8]. 16 hours post seeding, control neurons showed almost no neurite growth on myelin-substrates (Fig. 5 E). Conversely, PRG3 expressing neurons developed neurites in the presence of myelin (Fig. 5 E). One critical protein causing neuronal retraction inherent in myelin is Nogo-A/RTN4 [31]. We used the Nogo-A/RTN4-specific, axon collapse inducing protein sequence in the spreading assay (Δ20 peptide consists of aa position 544-725 in rat Nogo-A) [31, 32]. Neuronal cells were cultured in the presence of different Δ20 concentrations to determine whether Nogo-A/RTN4 can exert axonal retraction in the presence of PRG3. Concentrations of 3.2 μM of Δ20 reduced spontaneous outgrowth in control cells (Fig. 5 F, G). The Δ21 protein of similar molecular weight (aa 812-918 of rat Nogo-A), lacking the neurite growth inhibition activity was used as negative control (Fig. 5 F, G) [31]. PRG3 neurons spread on Δ20 substrate and formed filopodia and neurites comparable to Δ21 control substrate (Fig. 5 F, H). Interestingly, PRG3CT^MEM^ showed slightly longer neurites on Δ21 substrate compared to wild-type PRG3 expressing cells (Fig. 5 F, H). In contrast, Δ20 significantly reduced neurite length of PRG3CT^MEM^ neurons, where PRG3 full length expressing neurons were unaffected (Fig. 5 F, H). These findings suggest two separate functional domains for PRG3 with neurite growth promoting activity at the CT and a neutralizing activity for neurite growth inhibitors.

**Figure 6:**
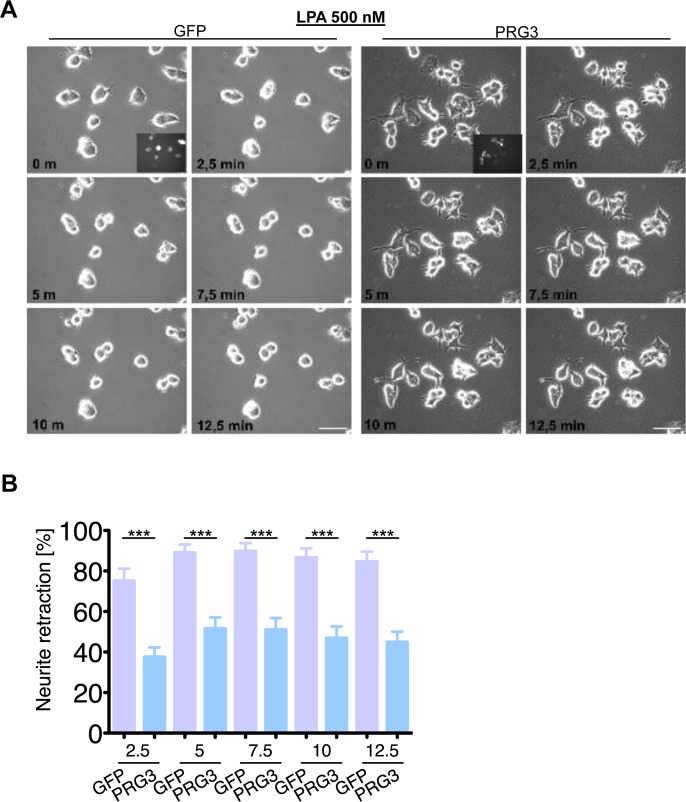
PRG3 impedes lysophosphatidic acid (LPA) induced axon collapse (**A**) Live time collapse assay with axon collapse inducing LPA. Representative time laps microscopy image series of GFP and PRG3 expressing neurons treated with 0.5 μM LPA. Transfection efficacy is shown in first image bottom right corner. Scale bar represents 50 μm. (**B**) Quantification of neurite length of neurons expressing GFP (control) or PRG3. Note, that GFP expressing neurons show significant higher number of retracted neurites and round cell bodies during the time lapse assay. Three independent experiments were carried out and differences were considered statistically significant with * p<0.05, ** p<0.01, *** p<0.001 (two-tailed *t-*test). Values are given as mean ± SEM.

**Figure 7:**
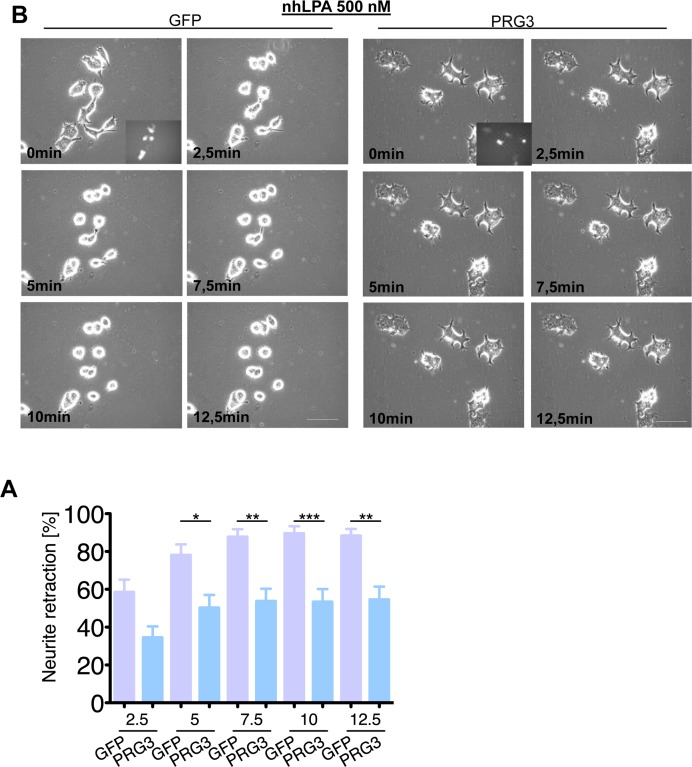
PRG3 impedes non-hydrolysable lysophosphatidic acid (LPA) induced axon collapse (**A**) Representative time laps microscopy image series of GFP and PRG3 expressing neurons treated with 0.5 μM nhLPA (non-hydrolysable lysophosphatidic acid, which is resistant to any ecto-phosphatase activity). Transfection efficacy is shown in first image bottom right corner. Scale bar represents 50 μm. (**B**) Quantification of neurite length of neurons expressing GFP (control) or PRG3. Note, that PRG3 expressing neurons are also resistant to the nhLPA collapse inducing activity. Three independent experiments were carried out and differences were considered statistically significant with * p<0.05, ** p<0.01, *** p<0.001 (two-tailed *t-*test). Values are given as mean ± SEM.

**Figure 8:**
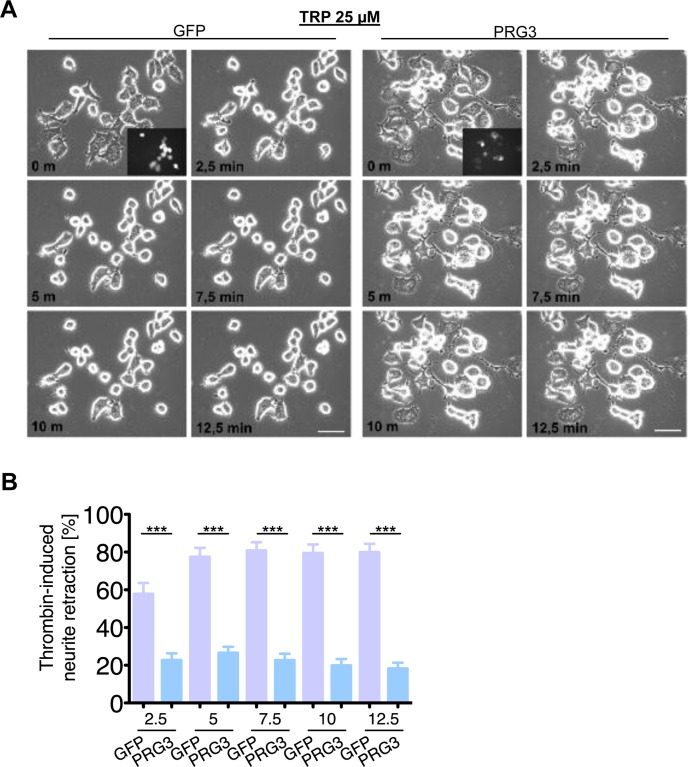
PRG3 impedes Thrombin-induced axon collapse (**A**) Representative time laps microscopy image series of GFP and PRG3 expressing neurons treated with 25 μM Thrombin (TRP). Transfection efficacy is shown in first image bottom right corner. Scale bar represents 50 μm. (**B**) Quantification of neurite length of neurons expressing GFP (control) or PRG3. Three independent experiments were carried out and differences were considered statistically significant with * p<0.05, ** p<0.01, *** p<0.001 (two-tailed *t-*test). Values are given as mean ± SEM.

We continued to investigate the neutralizing neurite growth inhibitory activity of PRG3 in time-lapse collapse assays. First, we investigated the potent, blood borne neurite growth inhibitor lysophosphatidic acid (LPA), which acts directly on RhoA via LPA Receptor (Kranenburg et al., 1999). Control and PRG3 expressing neurons were treated with 500 nM LPA. Approximately 90 % of all neurites initially displayed collapse in control neurons, whereas PRG3 expressing neurons retracted to 48% of their initial neurite length on average (Fig. 6 A, B). This effect could be induced via lipid phosphatase activity shown in PRG1 [33]. However, previous investigations revealed that PRG3 lacks detectable lipid phosphatase activity [22, 23]. Therefore, non-hydrolysable, phosphatase-resistant LPA (nhLPA) was used to verify these reports. The identical collapse assays were performed with nhLPA. Again, only control neurons contracted after 7.5 min comparable to native LPA controls, whereas PRG3 neurites also resisted nhLPA-induced neurite collapse (Fig. 7 A, B). General LPA receptor expression was unchanged after PRG3 expression (Fig. S5 C). Thrombin (TRP) represents another relevant blood borne neurite growth inhibitor. We treated controls and PRG3 expressing neurons with 25 μM TRP. Control neurites collapsed to over 80 % from their initial length within 5 minutes and displayed an overall rounded phenotype. In contrast, PRG3 neurites retracted by 30 % only and the neuronal cell shape was unaffected (Fig. 8 A, B). Thus, our data demonstrated that PRG3 attenuates the RhoA signaling cascade induced by common neurite growth inhibitors.

We tested calyculin (100 nM), a general cytoskeletal collapse-inducing compound, to exclude that PRG3 may inhibit overall cell motility (Fig. S5 D). Calyculin is a strong serine/thereonine protein phosphatase inhibitor and induces rapid contractile forces by depolymerizing actomyosin and microtubules. Following calyculin treatment PRG3 expressing neurons contracted to the same extent as control neurons, demonstrating that PRG3 expression does not impede general cytoskeletal dynamics and microtubule function (Fig. S 5 D).

### Increased axonal sprouting in PRG3 transgenic animals

**Figure 9:**
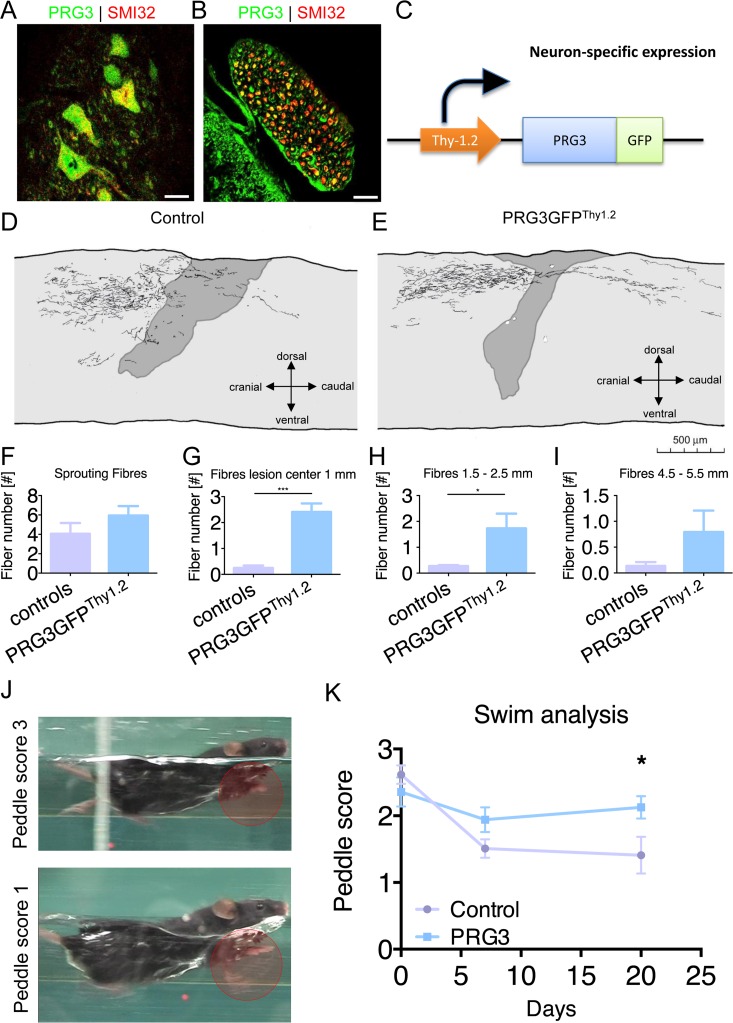
PRG3 is present in spinal cord neurons and contributes to spinal regeneration after spinal cord injury (**A-B**) Double-immunocytochemistry showing that PRG3 is co-expressed with the motor neuron marker SMI32 (non-phosphorylated neurofilament) in murine spinal cord motor neurons in the ventral horn (**A**) and their axons in the ventral root (**B**). Scale: 20 μm. (**C**) Schematic illustration of the Thy1.2 driven PRG3GFP transgenic mouse, cDNA construct of PRG3-GFP was subcloned downstream of the artificial Thy1 neuronal specific promoter, the construct was introduced as transgene in 129S zygotes. (**D-E**) Camera lucida reconstruction of spinal cord hemisection performed in control (n=4). (**D**) and PRG3 transgenic (n=3) (E) animals. (**F-I**) Sprouting fibres (**F**) and fibres 1 mm (**G**), 1.5 − 2.5 mm (**H**) and 4.5 − 5.5 mm (**I**) rostral to the lesion analysis of control and PRG3 transgenic animals after dorsal hemisection based on camara lucida reconstructions. (**J**) Representative image of the swim test score 3 and 1 display functional locomotion recovery analysis. (**K**) Swim test analysis. Baseline swim analysis was performed before dorsal hemisection (time point 0). After 7 and 20 days, swim performance was again scored. PRG3 transgenic animals show significant increased swim performance 20 days post spinal cord lesion compared to controls. Data plotted as mean ± SEM. Statistical differences were analysed by two way anova with Bonferroni post hoc analysis including repeated measurement correction (n = 5 per group). P value was set as * = p<0.05: ** = p<0.01; *** = p<0.001.

Our *in vitro* experiments identified PRG3 as a potential opponent of released myelin after spinal cord injury. Thus, we immuno-stained spinal cord sections for PRG3 and found spinal cord motor neurons and their efferent axons in ventral roots positive for PRG3 (Fig 9 A, B). We further assessed the potential of PRG3 to counteract axonal damage after spinal cord injury *in vivo*. For this we performed dorsal T-lesion at TH8 followed by axonal BDA tracer injection into the motor cortex in Thy-1.2 promoter driven (neuronal specific) PRG3-GFP transgenic mice (PRG3GFP^Thy1.2^, Fig. 9 C) [34]. Camera lucida analysis revealed neuronal sprouting 21 days post-operation in wild-type littermates and PRG3GFP^Thy1.2^ transgenic mice (Fig. 9 D, E). Increased axonal fibers were found 1 mm and 1.5 − 2.5 mm distal to the center of the lesion in PRG3GFP^Thy1.2^ mice compared to controls (Fig. 9 G, H). Importantly, no difference was found proximal to the lesion in both groups indicating equal BDA loading and fiber density proximal of the injury side (Fig. 9 F).

Hence, we investigated the functional impact of the increased axonal sprouting. Complex motor behavior was tested in spinal cord lesioned animals using a standardized Schnell-swim-test (SST). The test monitors compensatory balancing forelimb movements as a sign of impaired hind limb motor function (Schnell et al., manuscript in preparation). Initially, both groups showed comparable peddle scores in training sessions before the spinal injury. Following spinal cord lesion, we found significantly improved motor function in PRG3GFP^Thy1.2^ mice compared to control littermates (Fig. 9 J, K).

## DISCUSSION

Our report revealed PRG3 as a strong conductor for neurite growth *in vitro*, *in vivo* and following spinal cord lesion. PRG3 is an integral membrane protein with six trans-membrane domains and a carboxyl terminal tail located putatively in the cytoplasm. Although the PRG family consists of 5 sequentially highly conserved members, PRG3 showed the strongest effects on morpho-logy and induced dramatic morphological changes in neuronal cells resulting in a pronounced induction of actin-enriched protrusions and neurite growth.

### PRG3 during neuronal development

We started our investigations with the expression analysis of all PRG family members in brain and other organs. PRG1 and PRG5 expression was found in discrete regions of the adult brain. In contrast, PRG3 transcripts are strongly regulated in development, with high expression levels during early postnatal stages [35]. In the spinal cord, PRG3 expression was found in postnatal stages but was absent in the adult and aged spinal cord.

Interestingly, this time window is characterized by an overall high activity of structural neuronal plasticity, including enhanced neuronal outgrowth and wiring. It is tempting to speculate that PRG3 may play a role in these processes, especially when considering the challenged morphology induced by PRG3 when expressed in neurons. However, this interpretation awaits further functional data *in vivo*. Also, whether PRG3 regulates synaptic transmission as reported for PRG1 [33] needs to be further investigated. It has been reported that PRG1 affects spine density and synaptic plasticity in a cell-autonomous fashion via interacting with protein phosphatase 2A and subsequent β1-integrin activation [36]. These data have been found by investigating PRG1 deficient mice and further investigations will focus on the deletion of the *prg3* gene at the genomic level to open such studies *in vivo*.

Our results show that PRG3 accumulates at the tips of neuronal processes, to be located primarily in pre-synaptic end tips and growth cones of neurons. This points PRG3 towards a growth navigator in a neurite-growth inhibitory environment. Such inhibitory brain environments are in fact present from postnatal stages on into fully matured adult brains and in aged. Interestingly, in the brain PRG3 is distributed in neuronal layers where structural plasticity is still taken place [35].

### PRG3 forms filopodia and elongates neurite growth upstream of RasGRF1

When expressed, PRG3 drastically challenges cell morphology especially of neuronal cell types. Formation of fine and long filopodia and membrane protrusions with complex PRG3 positive branching was observed. Conversely, RNAi knock down of PRG3 impeded filopodia formation and neurite outgrowth resulted in neurite lengths below the level of controls [35]. These membrane protrusions are driven by cytoskeletal dynamics, which are to a large extent regulated by the small molecular weight GTPases of the Rho family [13, 37]. So far, the Rho family GTPase Cdc42 occupies a central position in regulating actin filament extensions and forming filopodia of different length and width [38, 39]. Within Cdc42 signaling events, activation of WASP and N-WASP by Cdc42 in the presence of PIP_2_ leads to ARP2/3 activation, which acts as a growth promoter for actin filaments, leading to the concept of ‘convergent-elongation’ model of filopodia formation [14, 40, 41]. However, the morphology of PRG3-induced filopodia resembled a phenotype also referred to hedgehog-like phenotype, which are in stark contrast to the short and thick filopodia characteristic for Cdc42 [22, 23].

Alternatively to Cdc42, Ras - Raf and MEK pathway was reported to induce neurite outgrowth [42]. This Ras pathway was critical for PRG3 mediated neurite formation. PRG3 full-length and PRG3CT induced filopodia outgrowth which was effectively reduced when the Ras mediator RasGRF1 was silenced. Our experiments further revealed that localization of PRG3 carboxy terminal domain is essential for operating as filopodia forming and growth-promoting. Remarkably, filopodia formation was rescued when expressing the membrane tagged carboxyl terminus. The function of the amino terminal six integral membrane domains needs yet to be determined, since plasma membrane targeting of the C-terminal domain rescued neurite growth activity. In contrast to PRG1, no ecto-enzymatic activity was found in the intracellular loops of PRG3 [23, 24]. Thus, the integral membrane domains may primarily serve as plasma membrane integrator and localizer. The topology renders PRG3 as a potential sensor, channel or receptor and therefore integral membrane domains are required for the exposure of putative extracellular binding domains to the outer surface.

We investigated the biochemical basis of the neuronal outgrowth phenotype by screening for consensus sequences of PRG3 C-terminus and other PRG's. Only two Ca^2+^ binding domains, CVVXNFKG and PXXESPLE were identified, whereas the rest of the 48 amino acid carboxy tail are disparate and do not give clear similarity hits to known protein domains. Noteworthy is a recent study reporting the interaction of PRG3/LPPR1 with other members of the PRG family [43]. Yu and colleagues reported that PRG1 facilitates the localization of PRG3 to the membrane and thereby promotes filopodia formation.

### PRG3 overcomes RhoA mediated neurite growth inhibition via PIP_2_ translocation

Filopodia formation and growth cone shaping are critically involved in axon growth control and are implicated in neuritogenesis [21, 44]. Knock down of PRG3 by RNAi impedes this process, pointing to an essential role of PRG3 in neurite formation [35]. Indeed, PRG3 is mainly localized at the plasma membrane and is enriched at the tips of filopodia and neurites. PRG3 expression is temporally restricted with highest levels in early postnatal stages, emerging in parallel to the repellent extracellular space at this developmental stage [2, 45]. We therefore asked whether PRG3 counteracts the inhibitory signals present during development and tested myelin-associated and various neurite growth inhibitors. Interestingly, PRG3 expressing neurites counteracted neurite-collapsing activities of myelin. Separate investigations of LPA, non-hydrolysable LPA (nhLPA) and thrombin revealed resistance to axon collapse and neurite retraction independently of the inhibitor used. These data indicate that PRG3 modulates a common downstream effector of myelin-associated growth inhibitors. Noteworthy, LPA receptors, Thrombin receptor and NgR, PirB/LILRB2 receptor signaling converge to the RhoA-ROCK-PIP5K kinase pathway eventually leading to actin depolyme-rization and neurite collapse [4, 28, 46]. It is tempting to speculate that PRG3 interferes at one level in this pathway and counteracts further downstream signaling in the conditions we used. Indeed, when PIP5K was overexpressed to reduce the available PIP_2_ pool, co-expression of PRG3 released the available membrane bound PIP_2_ pool resulting in filopodia growth [22].

**Figure 10:**
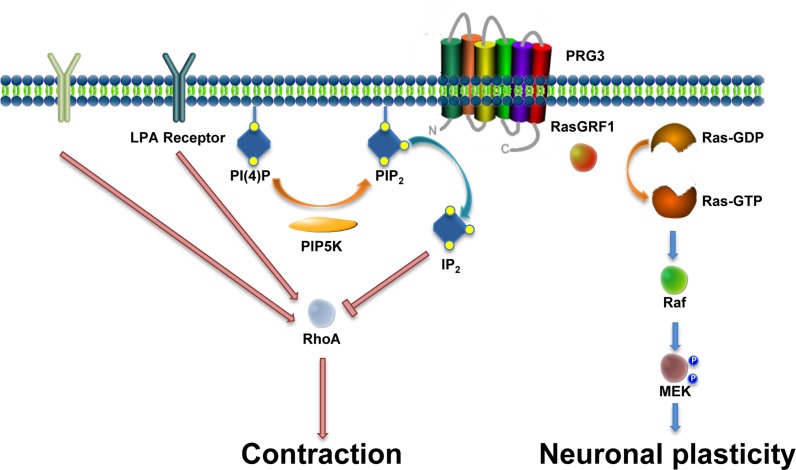
Scheme of the PRG3 action on RhoA-Rock-PIP5K and RasGRF1 signaling in axon growth and neuronal plasticity There is experimental evidence for two independent functions of PRG3. First, PRG3 interferes with the RhoA-Rock-PIP5K pathway through translocation of the lipid messenger PIP_2_. This central signal interference affects general downstream neurite growth inhibitors such as myelin-associated molecules such as Nogo-A, LPA and Thrombin. Secondly, interaction of PRG3 with RasGRF1 induces filopodia formation and neuronal plasticity via downstream effectors such as Raf and MEK.

Thus, these data are consistent with the notion that under neurite retracting conditions PRG3 interferes with the common RhoA-ROCK-PIP5K pathway and counterbalances the axon collapsing activity (Fig. 10). Whether this scenario also happens during different developmental phases in the brain is a topic of ongoing research. So far, we propose that PRG3 promotes filopodia initiation and neurite growth during the critical periods of CNS (brain and spinal cord) development and may assist in events of axonal fine tuning finalizing neuronal circuits. Moreover, PRG3 can interfere with signals coming from a repelling microenvironment, thus making this trans-membrane protein a valid candidate for promoting neuronal regeneration in the adult.

### PRG3 overcomes axonal repulsion *in vivo*

One urgent quest in a clinical setting is to promote spinal cord regeneration after traumatic spinal injury. We investigated the potential impact of PRG3 on spinal nerve regeneration, using transgenic mice over-expressing PRG3GFP^Thy1.2^ in neurons of the motor cortex. Following dorsal lesion of spinal axons in mice, PRG3 transected fibers showed increased sprouting distal of the lesion. Importantly, these sprouts are crucial for spinal cord regeneration [47, 48]. We performed locomotor function tests in PRG3GFP^Thy1.2^ transgenic animals and control littermates to evaluate the functional consequences of increased axonal regeneration. We found improved motor function in PRG3GFP^Thy1.2^ mice. These data demonstrate that PRG3 impedes neuronal growth restriction *in vivo* and fosters functional regeneration in the adult central nervous system.

In summary, PRG3 is an integral trans-membrane protein and is highly enriched at filopodia and neurite tips *in vitro* and pre- and post-synaptically *in vivo*. PRG3 expression promotes the extension of axons and its growth-promoting activity is linked to its carboxyl terminus. The plasma membrane localization of the PRG3 carboxyl terminus is pivotal for its function. This function is executed via a RasGRF1 dependent pathway for filopodia formation and axon growth. PRG3 prevents LPA, TRP and myelin-induced axon collapse via PIP_2_ membrane release.

In a clinically important setting we tested the role of PRG3 in spinal cord lesion. We could show that PRG3 promotes significantly axonal outgrowth and functional recovery *in vivo* after spinal cord injury. Essentially one of the challenges in CNS repair and therapy can be thought of as ‘turning back the clock’: promoting processes that occur during development but fail to or proceed inefficiently after maturation or injury. Here, we provide evidence that PRG3 may represent one neuronal component of such a developmental clock.

## METHODS

### Cell lines, transfection and immunoblotting

The rodent neuroblastoma cell line N1E-115 was obtained from ATCC/LGC-2397 (Germany) and was cultured under standard condition containing DMEM medium (Biochrom, Berlin, Germany) supplemented with 10% fetale bovine serum (Biochrom, Berlin, Germany), 1% Penicillin/Streptomycin (Biochrom, Berlin, Germany) and 1% Glutamax (Gibco/Invitrogen, California, USA). Cells were passaged at approx. 80% confluence by adding trypsin after 1 PBS wash step and incubated for 5 min, then centrifuged at 900rpm/5 min.) Cell lines were transfected according to Broggini et al [24]. Briefly, cells were plated at 20 000 /cm^2^ in 6-well plates and held under standard conditions. 24h after seeding transfection was performed using Roti-Fect (Roth, Karlsruhe, Germany) according to the manufacturer´s protocol. Western blot analysis was performed as described previously [49]. Cells were maintained under standard conditions. For protein extraction samples were lysed with NP 40 buffer containing a protease inhibitor cocktail (Roche, Basel, Switzerland) and homogenized by ultrasound (Bandelin Sonoplus, at 67 %). After 20 min on ice, samples were centrifuged at 8000 rpm for 8 min. Supernatants were measured with Nano-drop (Thermo-scientific, Massachusetts, USA). Samples were mixed with loading buffer (4x) and Reducing agent (10x) (Invitrogen, California, USA) and boiled at 96°C for 8 min. Equal amounts of protein sample were loaded on 4-12 % SDS-NuPage Gel (Invitrogen, CA, USA) and electrophoresis was performed in MOPS-buffer, transferred on PVDF membranes (Roth, Karlsruhe, Germany) and efficiency was checked with Memcode Stain kit (Thermo Massachusetts, USA) according to the user manual. Membranes were blocked in PBS containing 2 % Magic block and 10 % Top block (Lubio science, Lucern, Switzerland) for 1h before further processed. Antibodies were incubated overnight at 4°C in roller tubes, followed by secondary antibodies incubated at room temperature for 1 hour. Detection was performed with ECL plus kit (GE-healthcare, Solingen, Germany).

### Primary cell culture

Primary neuronal cortical cultures were prepared from E18 rat embryos and 7 × 10^4^ cells seeded on 12 mm glass coverslips coated with poly-L-lysine (0.1 mg/ml H_2_O) and cultured for 48 h in Neurobasal medium supplemented with 0.5 % GlutaMax, 0.02% B27 and 1 % Penicillin/Streptomycin (all from Gibco/Invitrogen). After two days of cultivation cells were processed for immunostaining essentially as described [50]. Briefly, cells were fixed by addition of equal volume of 4 % PFA directly into the medium for 15 min at 37°C. Afterwards, cells were washed in PBS and post-fixed for 20 min in 4 % PFA at room temperature (RT). Cells were permeabilized in 0.2 % Triton X-100, blocked with 5 % goat serum, 0.1 % BSA in PBS for 1 h and incubated with primary antibodies specific to PRG-3 (D43 clone, dilution 1:5000) and βIII tubulin (Covance, dilution 1:1000) were applied overnight at 4°C followed by washing steps in PBS and incubation with Alexa fluorophore-conjugated secondary antibodies. Images were acquired by confocal microscopy with an LSM510 microscope (Zeiss).

### Antibody generation and immunoblots

We designed peptide antibodies against PRG3 based on the protein sequence in the hydrophilic domains. The following peptide sequences were used: aa position 280-293 of the C-terminus of human PRG3: KGTQGSASKPKPED, aa position 296-310 (D43) of the CT of human PRG3: GVPLMAFPRIESPLE; aa position 186-199 of the third extracellular loop of human PRG3: LEVIEKARRSFPSK; aa position of the N-terminal domain of human PRG3: MAVEGNNTQRSYSIIP. Peptides were synthesized at the core facility of the Netherlands Cancer Institute (Henk Hillman, Amsterdam, The Netherlands). An amino-terminal cysteinyl residue, which is not part of the PRG3 sequence, was included for conjugation of the peptide to a carrier protein. Rabbits were immunized and the specificity of the serum was further tested on immunoblots and on tissue sections by blocking via peptide incubation prior to adding the antiserum. PRG3 antisera were used at a 1:2000 to 1:5000 dilution. Secondary anti-rabbit antibody coupled with horseradish peroxidase was used at a 1:5000 dilution, and visualized by incubation in ECL detection reagents (Amersham Pharmacia, Germany), and for equal loading controls a mouse β-actin monoclonal antibody (Sigma, Germany) was facilitated.

### Expression vectors and knock down vector cloning

RT-PCR was used for cloning of PRG3 from rat, mouse and human mRNA samples. For sequence alignments and homology searches of PRGs we utilized the www.ncbi.nlm.nih.gov database and A Plasmid editor software (ApE; MW Davis, Utah, USA). Trans-membrane domains have been predicted using the Kyte Doolittle algorithm and all orthologous sequences of PRG3 (human, mouse and rat) are deposited at the NCBI database (Human PRG3 GenBank accession no. AY304516; Rattus norvegicus PRG3 GenBank accession no. AY299399; Mus musculus PRG3 GenBank Accession no. AY345342). We amplified the fragments by PCR and cloned the resulting amplicons into the pEGFP (Takara, Heidelberg, Germany), and pCLEG (Stefan Schumacher, Ulm, Germany) vectors. C-terminal domain of PRG3 was cloned respectively. According to the criteria of Naito et al [51] two short interfering RasGRF1 RNAs were designed. Cloning of the synthetic oligonucleotides into the pSuperRFP vector (modified pS-GFPneo; OligoEngine, Seattle, USA) was performed by digesting the empty vector with Bgl2 and EcoR1 according to the manufacturer's instruction.

### Immunocytochemistry and histological analysis

Cells were plated 2500/cm^2^ in 12 well plates on coverslips and cultured under standard condition for several days. Cells were than fixed with 4 % PFA and immuno-stained for actin (Thermo scientific, Massachusetts, USA) overnight and DAPI (1:10000) for additional 5 min. Coverslips were mounted on slides with Immu-Mount (Thermo scientific, Massachusetts, USA). Pictures were taken with Olympus X71 microscope with x1000 magnitude. Exposure time was equal in different cell lines. Images were taken with cell- F software (Olympus, Tokyo, Japan). Image quantification was performed with CellProfiler 2.0 Software (Broad Institute). Animals were deeply anesthetized, followed by PBS and PFA (4%) perfusion. The brains were harvested and fixated in PFA o/n and dehydrated by a sucrose gradient (10%, 20% and 30%). After dehydration, the tissue was emerged in Tissue Tek (Sakura, Netherlands) and frozen at −20°C. Cryosections (20 μm) were obtained on coverslips and permeabilized using methanol. Slices were blocked with 1% Casein (Sigma, Germany) for 1 hour.

### Spinal cord and ventral root immunostaining

Deeply anesthetized mice were perfused with 4 % PFA, and spinal cords with attached ventral roots dissected, post-fixed overnight, and cryoprotected in 30 % sucrose for 2 – 3 days at 4°C. After tissue embedding, 20 μm transverse sections of the spinal cord were cut on a cryostat and blocked with PBS containing 0.1 % Triton X-100, 5 % goat serum, and 0.5 % BSA, on glass slides. Sections were blocked with 5 % goat serum, incubated overnight with primary antibodies specific to PRG3 and nonphosphorylated neurofilament (SMI32, Covance) including 0.5 % BSA, washed, incubated with fluoro-chrome-conjugated secondary antibodies, and mounted in Fluoromount [52]. Images were acquired by confocal microscopy with an LSM510 microscope (Zeiss).

### Electron microscopy

One 3-month old mouse, deeply anesthetized with a mixture of Dormicum and Hypnorm (15 μl/g body weight), was transcardially perfused with phosphate-buffered saline (PBS), followed by 150 ml of a fixative containing 4% formaldehyde and 0.05% glutaraldehyde in PB (0.1 M, pH 7.4) according to a protocol approved by the Veterinary Department of the Canton Zurich. Brain was postfixed for 1 h and, after several washes in PB, 100-μm-thick vibratome sections were obtained from the cerebral cortex and hippocampus. For pre-embedding immunoperoxidase method, sections were incubated in 10% goat serum in PBS for 1 h and incubated with 1 μg/ml of the antibody D43 in 2% goat serum/PBS for 2 days at 4°C. After several washes in PBS the tissue was processed for the avidin-biotin horseradish peroxidase complex method (ABC Elite; Vector Laboratories, Burlingame, CA, U.S.A.). Sections were washed in cacodylate buffer (0.1M) and osmicated in 1% OsO_4_ for 30 min, dehydrated in increasing concentrations of ethanol, and embedded in Epon resins. Ultrathin sections were imaged using a digital camera (Gatan 791 multiscan; Gatan Inc., Pleasanton, CA, USA) attached to an EM10C electron microscope (Zeiss, Oberkochen, Germany).

### *In vitro* live cell imaging

Cells were plated on 8 cm^2^ cell culture dish with a cell density of 4000 cell per cm^2^. Control cells were transfected with peGFP-N1. Cells were continuously exposed to the light and 38°C temperature during the experiment. Experiments were performed using Zeiss Axiovert 20 M (Zeiss, Jena, Germany), camera exposer time was set to 500 ms. Movies were acquired with Axiovision Software (Zeiss, Jena, Germany). In control peGFP-N1 transfected cells no morphological changes were found during 1 h. No morphological change was visible.

### PIP_2_ distribution in cells

For PIP_2_ distribution detection was performed as described [53]. Briefly cells were plated on coverslips and then transfected with a RFP fusion protein containing the PH domain of PLC1. This construct (RFP-PH^PLC^) functions as an in vivo probe for intracellular localization of PtdIns(4,5)P_2_. Twenty hours after transfection coverslips were mounted and cellular distribution of RFP-PH^PLC^ was examined by confocal microscopy. Quantification of the membrane- cytosol ratio for RFP-PH^PLC^ was performed with Image J.

### *In utero* electroporation

The *in utero* electroporation experiments were carried out as described [54], in accordance with a protocol approved by the local animal welfare committee. Briefly, we used time-pregnant mice at E15–E16 (post coitum). The uterine horns of anesthetized mice were exposed and the DNA solution was injected through the uterine wall into the lateral ventricle of two of the embryos. Five 38 V pulses of 50 ms were applied at 950 ms intervals by holding the injected brain with forcep-type electrodes connected to a square–pulse generator (CUY 21 Edit, Unique Medical Imada, Miyagi, Japan).

### Dorsal hemi section and anatomical analysis

Dorsal hemisection (T-lesion) in mice was performed as previously described [31]. Briefly, eight to seventeen weeks old PRG3GFPThy1.2 mice and wild-type littermates of both sexes (with 20 – 32 g body weight) were taken. Anaesthesia was performed with a subcutaneous injection of Hypnorm (120 μl / 200 g body weight; VetaPharma Limited, Sherburn-in-Elmet, UK), and Dormicum (0.75 mg in 150 μl distilled water per 200 g body weight; Roche Pharmaceuticals, Basle, Switzerland). Vitamin A eye ointment (Vitamin A Augensalbe®, Bausch & Lomb Swiss AG, Steinhausen, Switzerland) was applied to protect the eyes from dehydration during the operation procedure. A partial laminectomy was performed at the thoracic level Th8 and T-lesion was conducted. Two weeks later, the animals were injected with 1.2 μl of a 10% of the high-resolution, anterograde tracer biotin dextran amine. 10 days after the tracer injection, animals were sacrificed. The spinal cords and brains were dissected and histologically processed. On sagittal sections, the number of intersections of BDA-labeled fibers with a dorsoventral line positioned at defined distances distal from the middle of the lesion was counted at a final magnification of 400x. The obtained sum of fibers was normalized for interindividual tracing variability by relating it to the total number of BDA-positive corticofugal fibers (determined in six cross-sections of the medulla oblongata rostral the pyramidal decussation) for each animal. The entire evaluation was performed on number-coded, randomly mixed animals, i.e., without knowing the genotype of the animals. Statistical comparison was done with the unpaired Students two tailed t-test if not otherwise stated.

### Behavioral testing and Schnell swim test (SST)

Baseline swim measurements were conducted after three weeks of handling and training as previously described [47]. At this time point most of the animals had become used to the swimming task and had stopped the occasional use of their forelimbs. After experimental spinal cord injury, behaviour was assessed at weekly intervals. Swimming behavior was used to study differences in motor performance. Two examiners were needed: one person to handle the mouse and one person for camera operations. The setup for the swim test consisted of a rectangular Plexiglas basin with a mirror placed at the bottom of the pool to allow simultaneous recording of side and bottom view of the animal. Water temperature was always set at exactly 23˚C in order to keep swimming velocity constant. In weekly sessions, swimming was documented until three undistracted, unhesitant runs per mouse were obtained for reliable quantification. The digital video camera (Panasonic, NV-MX500EG, with constant shutter speed at 1/250 sec. and in sequences of 24 frames per sec.) was mounted on a trestle which allowed the filming parallel to the moving animal. After completion of the swimming sessions, video recordings were analyzed under slow motion or manual frame-by-frame option. Swimming sequences were transferred to a PC and de-interlaced using Pinnacle Studio Version 10 (Pinnacle Systems, Avid Technology, Inc., California) software with resulting in 24 fields/sec. The forelimb strokes were quantified in slow motion mode and the peddle score was calculated. The peddle score determined the total of 60 cm distance. This strategy revealed the smoothness of each run. The mean value of three undisturbed runs were chosen and the peddle score calculated: Score 1, bilateral foreleg strokes over long distance; score 1.5, bilateral foreleg strokes with partial gaps without bilateral strokes; score 2, single sided foreleg strokes over the total distance or at least half distance; score 2.5, random single foreleg strokes; score 3, no foreleg strokes. More details of the Schnell swim test are in preparation (Schnell et al., manuscript in preparation).

### Statistical analysis

Quantitative data from experiments were obtained as stated in the figure legend. Analysis was performed using unpaired two taled Student's *t* test if not otherwise stated (Excel, Microsoft, Seattle, WA, USA). Data from all experiments were obtained from at least three independent experiments. For survival analysis we used the GraphPad Prism software (GraphPad Software, Inc., LaJolla, USA). The level of significance was set at *p < 0.05. Error bars represent ± SD if not otherwise stated in the figure legend.

### Extended protocol

The extended protocol of the Schnell swim test will appear in a separate publication (Schnell et al., manuscript in preparation).

## SUPPLEMENTARY MATERIALS FIGURES


