# Supplementary material for: Plasticity Related Gene 3 (PRG3) overcomes myelin-associated growth inhibition and promotes functional recovery after spinal cord injury
Source: Aging (Albany NY). 2016 Oct 15;8(10):2463–83. doi: 10.18632/aging.101066 (PMC5115901; doi:10.18632/aging.101066)
Supplement: Supplementary file 1 [file aging-08-2463-s001.pdf]

SUPPLEMENTARY MATERIAL

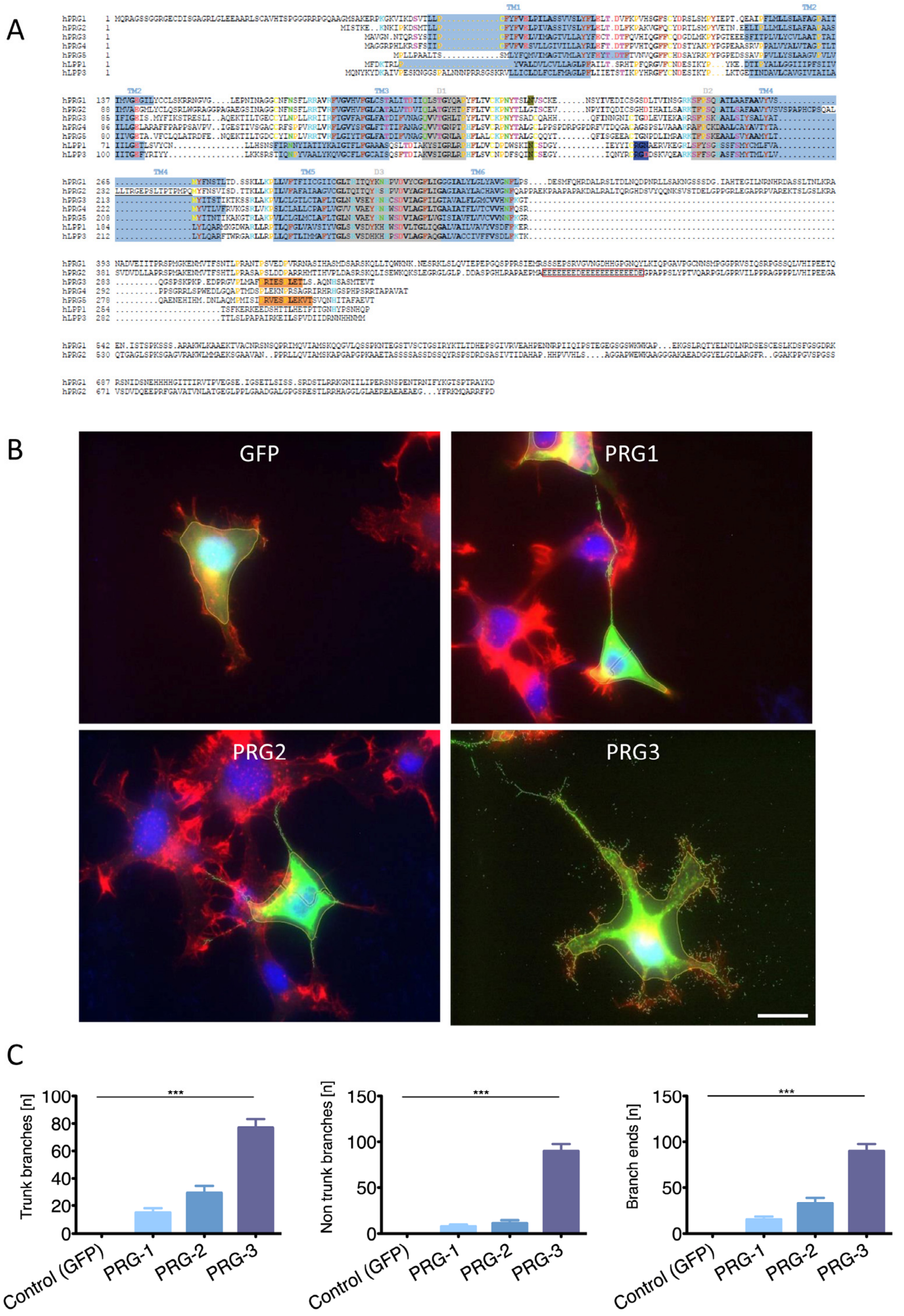

Supplementary Figure S1. Human PRG1 - 5 amino acid sequence alignment to human LPP1 / LPP3 and comparative analysis of PRG1, PRG2 and PRG3 in neuronal cells. (**A**) Amino acid sequence of human PRG1 to 5 (GenBank Accession no. NM_014839.4, NM_024888.2, AY304516, NM_022737.2, NM_001037317.1) aligned to human LPP1 and 3 (NM_176895.2, NM_003713.4). Putative transmembrane domains TM1 - TM6 are highlighted in blue. (**B**) Representative images of control GFP, PRG1, PRG2 and PRG3 transfection in N1E-115 cells. (**C**) N1E-115 control GFP, PRG1, PRG2 and PRG3 number of trunk branches, non trunk branches and branch ends comparison. Only PRG3 shows significant different phenotype. Three independent experiments were carried out. Differences were considered statistically significant with values mean ± SEM (one way anova, Dunnett's multiple comparison test * = $p<0.05$, ** = $p<0.01$ *** = $p<0.001$).

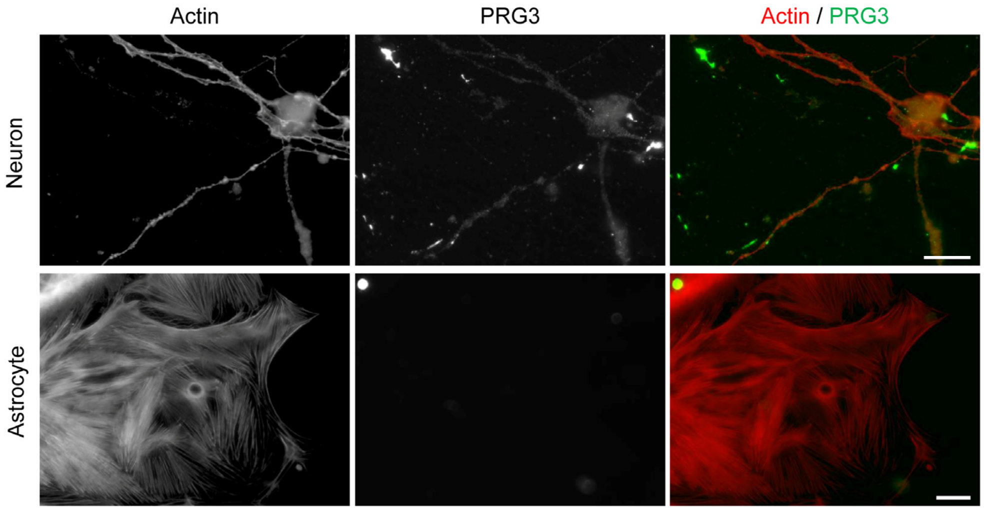

Supplementary Figure S2. PRG3 and actin staining in primary cortical neurons. Primary neurons were stained positive for PRG3 in primary mixed neuron-astrocyte cultures. In comparison, astrocytes were negative for PRG3 immunostaining (Scale bar Neuron = 20µm, scale bar astrocyte = 50 µm).

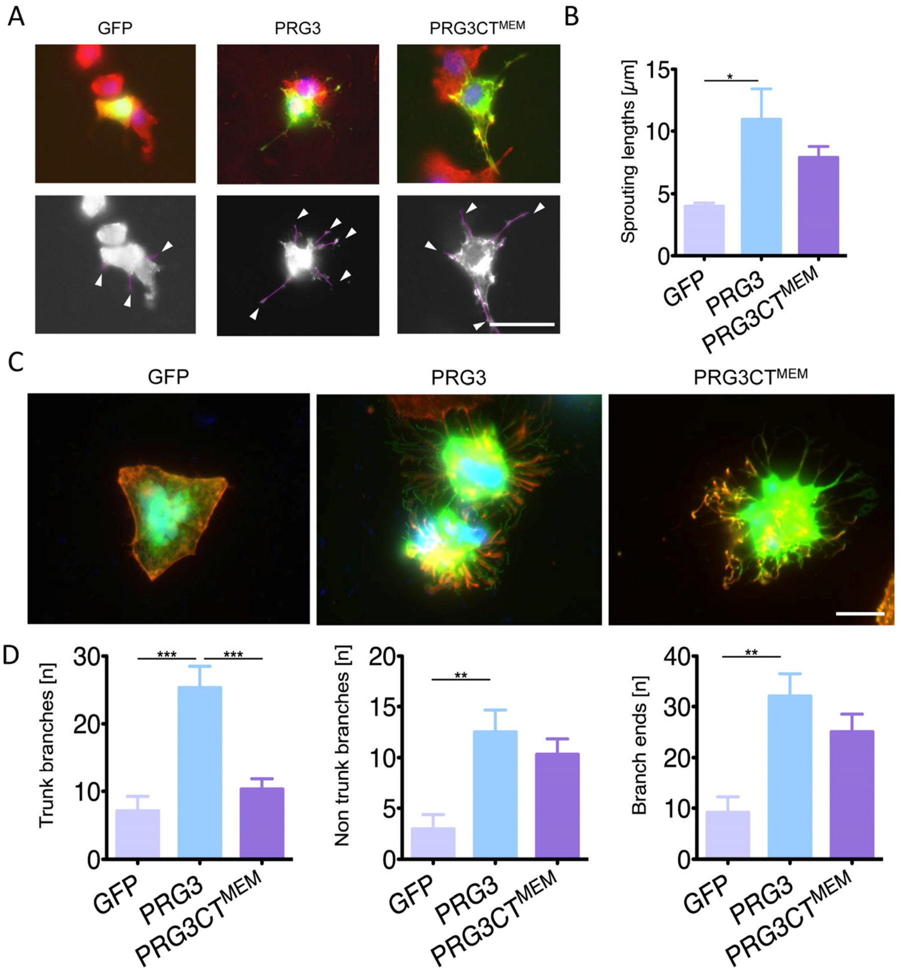

Supplementary Figure S3. PRG3 promotes neurite-like sprouting and hedgehog-like morphology in non-neuronal cells. (**A**) P19 embryonic carcinoma cells expressing PRG3 and $PRG3^{MEM}$ show long membrane protrusions (upper row). Traces of outgrowing protrusions for quantification are shown in bottom row (arrows). Scale bar represents 10µm. (**B**) Quantification of neurite-like membrane protrusions (sprouting length) in GFP, PRG3 and $PRG3CT^{MEM}$ expressing P19 cells. Three independent experiments were carried out and differences were considered statistically significant with values mean ± SEM (one way anova, Bonferroni correction, multiple groups comparison; * $p<0.05$, ** $p<0.01$, *** $p<0.001$. (**C**) PRG3 and $PRG3CT^{MEM}$ induce hedgehog-like morphological phenotype in HeLa cells. Representative images of GFP transfected cells (GFP), PRG3 overexpressing HeLa cells (PRG3) and membrane targeted C-terminus of PRG3 ($PRG3CT^{MEM}$). Scale bar equals 10 µm. (**D**) Quantitative analysis of hedgehog-like extensions in GFP, PRG3, and $PRG3CT^{MEM}$ expressing HeLa cells. Measurements of trunk branches (close to cell bodies), non-trunk branches (peripheral branches) and tip branches are given. Statistical analysis was performed with one way anova (Bonferroni correction for multiple comparisons from three independent experiments); * $p<0.05$, ** $p<0.01$, *** = $p<0.001$. Error bars are given as ± SEM of each group.

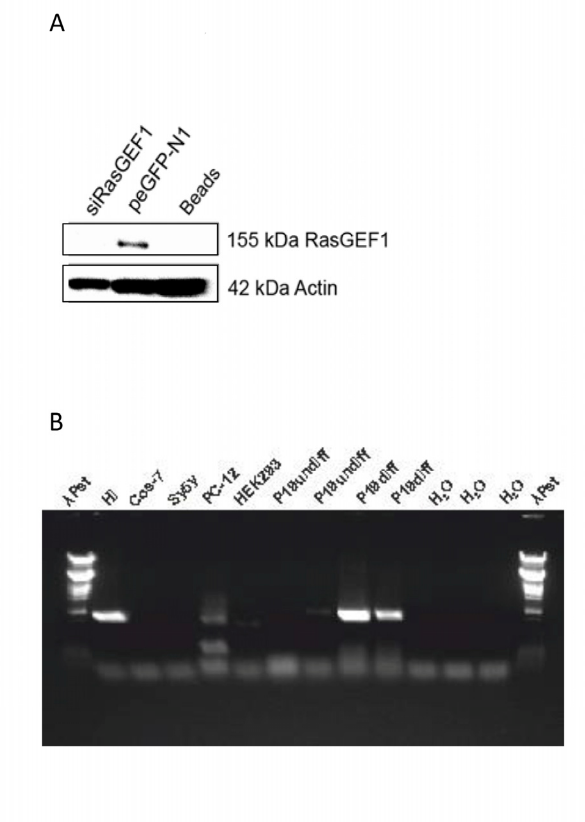

Supplementary Figure S4. RasGRF1 siRNA knockdown control and cell line PRG3 expression profiling. (**A**) Control RasGRF1 immuno-pulldown shows effective RasGRF1 knockdown after transfection (**B**) mRNA expression of PRG3 in different cell lines, hippocampal tissue (Hi) served as positive control.

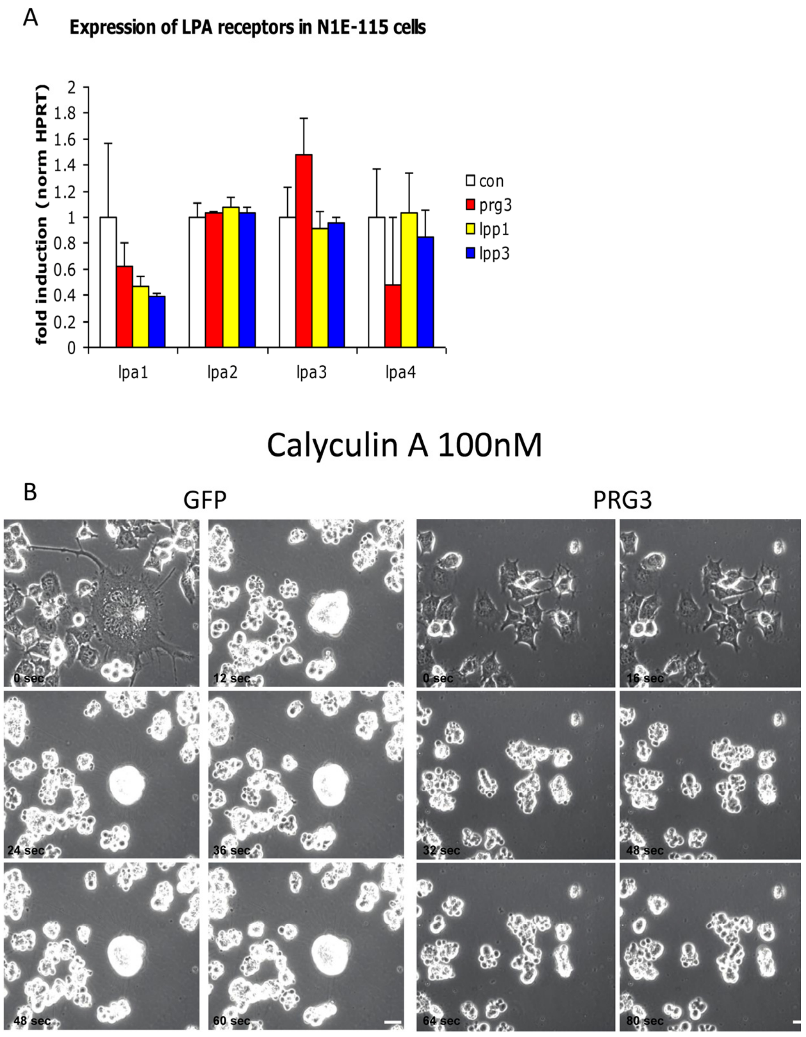

Supplementary Figure S5. N1E-115 LPA receptor expression and Cell contraction control using calyculin A. (**A**) Expression analysis of PRG3, LPP1, LPP3 and control N1E-115 cells shows expression of all 4 receptors. (**B**) Representative time laps microscopy image series out of three independent experiments are shown of 100nM Calyculin A induced cell collapse in GFP and PRG3 cells after 80 seconds. Scale bar represents 50µm.
